# Collaborative risk governance in informal urban areas: The case of Wallacedene temporary relocation area

**DOI:** 10.4102/jamba.v9i1.386

**Published:** 2017-04-13

**Authors:** Patricia J. Zweig

**Affiliations:** 1 Department of Geography and Environmental Studies, Stellenbosch University, South Africa

## Abstract

Community-based disaster risk management (CBDRM) is an emancipatory approach that aims to empower local communities in reducing their own risks. A community risk assessment (CRA) is an essential element of CBDRM, incorporating highly participatory processes of hazard identification and vulnerability analysis. By incorporating local knowledge and insights, together with those contributed by other external role players, the nature of local risks can be more accurately identified, giving consideration to their causal factors, the nature of their realised impacts or potential effects on a local community and the challenges posed in addressing them. Reflecting on the process and outcomes of a CRA conducted in an informal settlement in the Cape Town metropolitan area, this article describes how one such risk assessment contributed to building local agency through a process of collaborative engagement. Offered as an example of possible best practice, it illustrates both the immediate and potentially longer term benefits to be derived from such a collaborative process, suggesting that a community-based risk assessment may contribute significantly to building more resilient communities. It concludes with a consideration of the challenges of sustaining longer term risk reduction efforts.

Everyone has the right to live in a great place. More importantly, everyone has the right to contribute to making the place where they already live great. (Kent 2013:n.p.)

## Introduction

The *South African Disaster Management Act* (Act 57 of 2002) is globally acknowledged to be a progressive piece of disaster management legislation. One of its strengths is that it explicitly foregrounds the importance of civil society in contributing to disaster risk management, building on a constitutionally driven imperative that encourages citizen participation in order to deepen democracy (South Africa 1996). Community-based disaster risk management (CBDRM) is an emancipatory approach to risk management that aims to empower communities in reducing local forms of risk and underlying vulnerabilities, thereby building more resilient communities. Section 2.1.4 of the South African Disaster Management Framework (South Africa 2005), the legal policy instrument that informs the implementation of the *Disaster Management Act*, recognises the value of collaborative risk reduction, explicitly stating that risk assessments should ‘actively include the participation of vulnerable communities and households’ (DMF 2005:62).

A community risk assessment (CRA) is an essential element of CBDRM, incorporating highly participatory processes of risk identification and analysis. Conducted at a relatively small geographic scale, a community-led risk assessment can best establish the nature, spatial extent, temporality as well as the lived realities of local risks and associated vulnerabilities. It also offers a ‘practical bridging strategy to integrate local development efforts on one hand with strategies to reduce the impact of priority disaster risks on the other’ (Holloway & Roomaney 2008:18). By incorporating local knowledge and insights, a CRA can identify the nature of local risks, giving consideration to a multiplicity of hazards, their realised impacts or potential effects on a local community. As Corburn (2002) suggests:

by relying on local contextual information for key data inputs, the assessment process may shift the risk discourse away from an expert-only model of analysis and decision-making towards a more democratic model. (p. 451)

Risk information derived from community participation can more robustly inform the setting of specific integrated development planning (IDP) objectives to reduce risks over time, allowing as Kent has stated above for people ‘to contribute to making the place where they already live great’ (Kent 2013:n.p.).

The tools employed in community-based risk assessments have been developed over several decades and were originally used by development practitioners, adult education specialists and various others, generally in rural contexts. They have been used, for example, in conducting Participatory Rural Appraisals (Chambers 1994), Rapid Rural Appraisals (Chambers 1981; McCracken, Pretty & Conway 1998) and Participatory Action Research (Kindon, Pain & Kesby 2008), terms that are often used interchangeably. The participatory methods employed in these and other similar processes are highly adaptable and can be employed in almost any context, more recently modified for use in urban environments (Moser & Stein 2011) such as informal settlements (Holloway & Roomaney 2008). The tools used are very simple and highly visual, making them particularly useful when working with poorly educated people, even those who are illiterate, allowing everyone to contribute in some way to the process. In particular, they encourage less privileged, marginalised local participants to contribute their views, slowly developing their self-confidence and a sense of their own agency in the process.

Although CRAs have become accepted elements of CBDRM (Mercer et al. 2008; Pelling 2007; Van Aalst et al. 2008), their inherent potential to deliver detailed information about local context is often missed because, when used extensively, they can become reduced to very standardised and formulaic ‘box-ticking’ exercises, more closely resembling typically more favoured top–down processes that rely on ‘non-objective value judgements’ (Corburn 2002:455). As such they fail to deliver the level of detail and nuance about local risk conditions for which they were intended. As a field-based community-driven participatory process, however, a CRA has the potential to reveal how area-based hazards and local vulnerabilities are perceived and experienced by local residents themselves. This is critical, because the perceptions of local residents often differ from those of outsiders to their communities (Cannon 2008; IFRC 2014; Wisner 2016). Driven by ‘different rationalities’ (IFRC 2014:8), local residents often prioritise risks quite different from those outsiders anticipate.

Supporting the principles of transparency, accountability and ethical practice, the benefits of a CRA should accrue directly to the communities who collaborate in the process. However, if risk reduction efforts are to effectively build resilient communities of the future, they should be appropriately designed for local context, especially if they are to achieve local traction. The uniqueness of each and every landscape should be comprehensively considered, from its particular physical attributes, such as topography, weather, climatic variability and infrastructural provision, to more social aspects of the environment, including historic and current institutional, political, socio-economic, cultural and even religious factors.

At the University of Stellenbosch, the Research Alliance for Disaster and Risk Reduction (RADAR) regularly conducts a 1-week field-based training course for local government officials, researchers, development practitioners and even local councillors, teaching the process of community-based risk assessment. Although essentially teaching ethical grounded practice to trainees, the aim of each community encounter is also to ensure developmental gains for the community involved in the collaboration. For over a decade, RADAR trainings have resulted in a variety of developmental and risk-reducing outcomes, from those that are quite visibly or physically manifested to less tangible benefits, such as community-led advocacy and strengthened community leadership.

Reflecting on the process and outcomes of a CRA conducted in an informal settlement in the Cape Town metropolitan area, this article illustrates the immediate and potential longer term benefits that may be derived from a robust community-based risk assessment process. Although providing experiential learning opportunities and professional skills development for trainees, the process derived benefits to both the community and the local authority role players who were involved. The article describes the nature of these successes and how they were achieved, concluding with an exposition of the typical challenges of sustaining longer term risk reduction efforts into the future.

## Evolving risks in urban Africa

More than a third of the world’s population are currently living in urban areas (United Nations 2014), whereas half of the people of Africa are anticipated to be urbanised by 2030 (Ruhiiga 2013; UN-Habitat 2010). Given the rapidity of this urbanising process, resulting from natural urban population growth, large-scale human movements into urban areas (migration) as well as the gradual transformation of once rural areas into urban spaces, the nature of disaster risk in urban areas is evolving simultaneously (Adelekan et al. 2015; Pelling 2003). Patel et al. (2015) suggest that risk is in fact ‘urbanising’ with urban communities becoming increasingly exposed to a range of hazards, both those that are naturally triggered and, increasingly, those that are human-induced (Blaikie et al. 2014). Contemporary urban ‘riskscapes’ are presenting ‘highly complex environmental, social and economic challenges’ (Vogel et al. 2016:515), necessitating a more integrated approach to risk reduction, particularly in the global South, which is most challenged to deal with what Pieterse terms the ‘urban poly-crisis’ (2008). This demands effective urban risk management to address risks that have become ever more complex and convoluted, necessitating consideration of their cumulative effects (Corburn 2002), and linkages to broader external processes across multiple scales – from the global to the local (Adelekan et al. 2015).

African urban spaces, however, are rich in nuance and texture, ‘profoundly shaped by intertwined processes of formalization and informalization’ (Ernston, Lawhon & Duminy 2014:1573), posing both developmental challenges and opportunities for innovative ‘home grown’ solutions. In the context of South Africa, there is, therefore, an imperative to ‘engage with a development agenda that includes the politics of informality and the complexities of social as well as environmental change’ (Ziervogel et al. 2016:1). The *South African Disaster Management Act* acknowledges the importance of a multi-sectoral approach and the pivotal role of disaster management in coordinating both pro-active actions and post-disaster response and recovery (Ziervogel et al. 2016). However, in order to achieve sustained risk reduction outcomes, members of affected communities should be involved in identifying the risks and in seeking appropriate solutions themselves. This has been reiterated by the UN Secretary General, Ban Ki Moon, in a recent summary following the World Humanitarian Summit in May 2016, in which he stressed that contemporary challenges can only be addressed through collaborative action involving a range of actors including those from civil society (United Nation 2016).

The next section of this article illustrates how the involvement of ordinary citizens from an impoverished informal settlement in Cape Town, South Africa, as a consequence of their involvement in a CRA process, achieved significant developmental and quality-of-life gains. The story highlights how a truly participatory problem-solving process brought a small community together with many sectors of a large metropolitan authority to address local risks, and in the process changed the level of community agency and prompted further collaboration.

## Introducing the Wallacedene temporary relocation area informal settlement

The small informal settlement of Wallacedene temporary relocation area (TRA) is situated within the larger suburb of Wallacedene, a sprawling suburban township located some 40 km from Cape Town in the eastern suburbs. The history of development in Wallacedene is inextricably linked to shifts in local government administration (Muzondo et al. 2004). Originally, falling under the jurisdiction of the Cape Divisional Council, administration of the area changed significantly in 1996 when the Cape Town metropolitan area was divided into six municipalities, with Wallacedene becoming part of the Oostenberg Municipality. In 1998, the Municipality forcefully evicted several hundred Wallacedene residents who had invaded private land, resulting in a landmark South African Constitutional court battle. Commonly referred to as ‘the Grootboom Case’ (Government of South Africa 2000; Huchzermeyer 2003), after housing activist Irene Grootboom who confronted the local authorities claiming constitutional rights to land and basic services, this marked a significant moment in South Africa’s political history and the evolution of citizen rights. It also served, inadvertently, to perpetuate a spotlight on Wallacedene, ensuring that the area continues to be the focus of political manoeuvring that has to some extent shaped its development history.

In 2002, the six individual municipalities were merged to form a single metropolitan entity, the City of Cape Town. Control of the City initially shifted several times from one political party to another, so that lack of constancy undermined local development, with each administration reversing the decisions taken by the previous entity. For this reason, during the early development of state-built housing programmes in Wallacedene, the City had not yet developed a clearly defined housing allocation policy, resulting in the development of an ‘unofficial’ informal allocation process controlled by the South African National Civic Organisation (SANCO). This resulted in a very loose correlation between official records and actual occupation (Whittal et al. 2004). When the City later tried to institute a formal process, it clashed head-on with an already well-entrenched and community-endorsed informal system, a legacy that to some extent informs the tenuous relationship Wallacedene has with the City today.

Low-cost housing development in South Africa, although a stated national development imperative, cannot keep pace with the growing demand (Huchzermeyer 2001; Thring 2003; Zweig 2015). In the City of Cape Town, for example, where the need for state-built housing has grown to around 300 000 households/units, it is only possible to supply 9000–10 000 new low-cost houses a year. The process of housing formalisation in crowded informal housing areas results in reduced housing density (Meth 2015), creating a surplus of people who do not receive houses left with nowhere to live. During the phased building of houses in Wallacedene, a TRA was established to accommodate people waiting to receive formal housing whilst bulk infrastructure was installed. Although, as the name suggests, the TRA was only intended to temporarily accommodate housing beneficiaries, once formal housing had been provided and the first occupants moved out, the area was immediately occupied by those marginalised by the de-densification process who sought new living spaces. Despite winter flooding, people continued to set up home in the TRA, constructing dwellings in what was essentially a wetland area. Recognising the inevitability of continued occupation of the TRA, the City eventually attempted to level parts of the settlement, installing elementary drainage to alleviate flooding. Due to the gradient of the land and its wetland character, however, this failed to adequately prevent flooding. Today, the Wallacedene TRA is considered by the City authorities to be a high-risk area, prone to seasonal flooding, repeated fire outbreaks whilst, because of unsanitary living conditions that have developed there, public health risks undermine daily life.

In 2015, in consultation with disaster management and community leaders, the TRA settlement was selected for RADAR’s forthcoming risk assessment training. At this time, a new leadership structure had just emerged, replacing the previous leaders who had been affiliated with a powerful local branch of SANCO, a civic organisation aligned with the ruling party. For SANCO, the TRA served as an example of how the City, ruled by the national opposition party, was failing poor marginalised settlement dwellers. This political dynamic had resulted in a stale-mate that undermined development in the area for many years. The new leadership structure, however, determined to remain apolitical in order to realise developmental change, and agreed to work with RADAR in coordinating community involvement in a participatory risk assessment process.

## Scoping the local risk context

Understanding local context is critical to deriving maximum benefits from a risk assessment process (Corburn 2002; Gibbs 1994). It is essential to undertake quite extensive background research to inform a comprehensive understanding of a particular local landscape and how it has changed over space and time. This involves a preliminary desktop review of existing information, including official government documents and published reports, disaster incident records, press statements and newspaper articles, particularly those from local newspapers, which often record the perspectives of local residents. In the South African context, a review of IDPs can also identify key area-based development issues, imminent planned developments and longer term spatial planning.

In advance of the field-based phase of a risk assessment, it is also important to interview key local stakeholders. Typically, these include local authority officials, community leaders, community-based organisations and various other local and external role players who are associated in some way with the community, possibly even the private sector, to enable an informed understanding of what might be quite varying perspectives. Transect walks with members of the community provide further context, affording opportunities to meet with other local residents and to explain the purpose of the intended risk assessment process to follow.

Thus, a scoping exercise involves significant relationship building ahead of the field process, establishing a measure of trust. It may also reveal some of the underlying, yet often unspoken, tensions in a community. Thorough preliminary scoping as described here helps to avoid asking inappropriate and ill-informed questions and also facilitates entry into a community, shaping the design of a risk assessment process to ensure that it is appropriate for the dynamics of a particular community.

## Assessing risk in Wallacedene

Over a brief 2-day period, in July 2015, following several days of classroom-based training in risk assessment methodology, learning about participatory methods and the ethics of working with communities, trainees spent time in the field, immersed in the real-life context of an informal settlement .Working together with residents from the Wallacedene TRA, their task was to interrogate the nature of risks prevalent in the settlement, developing an insightful understanding of the hazards faced by residents, the nature of their vulnerability to these hazards, the coping mechanisms and any longer term adaptations they might have developed in response to the threats they live with.

The field work commenced with a 1-day workshop in which trainees employed simple participatory methods and highly visual tools such as problem trees and community mapping exercises, to identify and prioritise the risks prevalent in the TRA community. The workshop elicited significant discussion and debate, revealing what community members perceived to be critical local risk and development issues. The process provided community members, perhaps for the first time, with an opportunity to consciously reflect on their existing living conditions, to begin to visualise an alternative and to recognise their own potential agency in eliciting positive change.

The workshop, attended by more than a dozen volunteers from the community who had been solicited by the leadership structure, assisted the trainees in identifying and understanding the nature of local risks. Once identified, the risks were then discussed at length with community members, who described in detail their experiences of living with them. It is important to understand that, with often limited vocabulary, informal settlements dwellers often describe risks metaphorically and seldom in more direct and obvious terms. The process is, therefore, slow and sometimes protracted, but eventually results in a shared understanding of the issues. These are later interrogated further and verified during transect walks through the settlement, both through observation and by conducting unstructured interviews with other residents. It is important to verify information in this way, particularly because workshop participants are often selected by powerful community gatekeepers, which might introduce bias, with workshop participants driving hidden leadership-led agendas. Field-based interviews ensure that the perceptions and opinions of a broader sample of residents are collected, also enabling the triangulation of workshop findings.

On the second day of the field-based risk assessment process, trainees undertook transect walks through the Wallacedene TRA settlement, meeting up with the workshop volunteers from the previous day, amongst whom there was now a visibly heightened level of enthusiasm. The volunteers were tasked to lead the transect walks along routes that they themselves determined, pointing out issues that had been described during the workshop. Their presence and those of the trainees, who were now provided with large aerial photographs of the settlement and hand-held Global Positioning System (GPS) devices, encouraged the participation of many other curious community members who volunteered additional information. Local community members were tasked to identify the location of their homes on the aerial images and, once they had orientated themselves in this way, were able to identify flood-prone areas, crime hotspots and the spatial extent of past fire events, also volunteering other spatial information, which was duly marked on the ‘map’.

The transect walks were followed by a discussion session to which local officials representing various sectors from the City and the provincial authorities had been invited. The ward councillor and other important local role players also attended. Making reference to the diagrams and charts generated during the workshop that were then exhibited on the walls, community members themselves began the session by presenting the findings of the risk assessment to the assembled role players. The floor was then opened for a university-facilitated multi-stakeholder discussion. As an external entity, the university was able to create a neutral space not often afforded to government functionaries, in which issues could be respectfully discussed and debated without the vitriol that might ensue if the local authority or a political figure had scheduled the meeting. Whilst community members were able to explain the challenges of living with the risks they had identified, local government officials similarly explained the challenges they faced due to bureaucratic processes and funding constraints. In this open forum, plausible solutions were discussed, described and agreed upon. Commitments to ongoing collaboration were made, and there was a mutual sense of achievement when the meeting finally adjourned.

## Reducing risks in Wallacedene: Collaboratively driven outcomes

In the weeks that followed the CRA fieldwork process, a report recording the findings and outcomes of the risk assessment was drafted by the university coordinator and circulated to all those who has been involved, including the trainees. It not only documented the findings of the risk assessment but also recorded critical points made during the discussion session, mutually agreed-upon solutions and proposed risk-reducing interventions. The widely disseminated report ensured that both the community and the City officials adhered to their committed actions in the weeks ahead.

As a result of the risk assessment, many risk-reducing interventions were undertaken that contributed markedly to improving the living conditions of more than 4000 people estimated to be living in the Wallacedene TRA. Initiated by the City’s Solid Waste Department, a large-scale clean-up programme was carried out. Previously, the lack of cooperation from the TRA community has made coordinated action impossible. Now, however, led by community leaders, illegal electricity lines were temporarily disconnected by community members themselves to facilitate the entry of bulldozers and waste collection trucks so that the accumulated waste could be removed in a large-scale operation. Thereafter, truck-loads of clean sand were delivered, and community members of all ages became enthusiastically involved in distributing it across the settlement. The City Health Department then planned and executed a rat-eradication programme, which they had committed to during the stakeholder discussion session, distributing rat boxes throughout the settlement.

As a result of these two initial interventions, there was a significant reduction in health risks in the settlement, whilst in an effort to sustain the cleaner conditions, members of the community began to monitor the indiscriminate dumping of waste themselves, enforcing stringent new rules amongst settlement residents. For example, the license numbers of vehicles dumping waste in the settlement are now visually captured on cell phones and transmitted to the City’s Law Enforcement Department, who are able to trace perpetrators, thereby discouraging further incidents. The City’s Health Department later coordinated the removal of dozens of diseased dogs from the settlement by an animal welfare organisation. This process was enthusiastically supported by community members, who assisted in identifying problem dogs and delivering them to awaiting officials, who either took them away for merciful euthanasia or provided advice on pet care. The Water & Sanitation Department installed new blocks of flush toilets to replace the broken ones identified during the risk assessment, employing local labour from the settlement. The local police commander, concerned to improve the strained relations between the community and the police service, responding to a creative suggestion made by a community leader, designed and printed calendars displaying critical emergency numbers. These were distributed to households throughout the settlement. Wallacedene TRA has recently received the provision of electricity. Working with the City authorities and Eskom, the national electricity service provider, the leaders successfully coordinated the shifting of dwellings and the widening of paths within the settlement to accommodate the new electrical tracks.

The immediately visible improvements and significant reduction of a variety of risks inadvertently strengthened the credibility of the fledgling leadership structure. Emboldened by their success, the TRA committee built a small office in the settlement from which to coordinate further actions. It has subsequently become an important place on the settlement landscape, a multi-purpose hub that bears testimony to the sense of community agency that has developed.

The risk assessment report became a valuable action document holding all parties to account and will hopefully continue to drive development interventions that have not yet been actioned, for example, with regard to fire and flood risk which have yet to be addressed. Recording the nature of existing risks and living conditions at a point in time, the report has contributed a base line of information against which change over time can now be measured. Uniquely focused on a single settlement, it is a valuable tool for the community, the ward councillor, as well as government functionaries and potentially various other external role players for whom it can be considered a blueprint for action.

## Success factors and challenges to sustained engagement

It is critical to understand why the CRA process was so successful in reducing risk in the Wallacedene TRA. There are several key factors to consider. Firstly, the presence of a new and dynamic leadership structure with a vision of a better future for the settlement, that was apolitical and open to working with outside stakeholders, allowed for both the coordination of activities and the building of trust amongst members of the TRA community. Secondly, City officials were prepared to listen, open to finding mutually acceptable solutions and, critically, were of an adequate level of seniority to authorise promised interventions^1^. Experiencing an immediate improvement in their quality of life, the community gained a sense of their own agency, providing the new leadership structure with a mandate to continue to drive and coordinate change on behalf of the community. Thirdly, the university, as an essentially neutral and outside entity, functioned initially as a mediator between the community and the local authorities, helping to broker conversations and build relationships, encouraging continued interaction.

Although participatory assessment has been shown to effectively reduce risk, there are without doubt also many challenges to achieving and sustaining successful outcomes. Although the remarkable changes that have been described here were achieved within less than a year, over recent months the pace of change has slowed, and communication between the City and the community has become less frequent, perhaps inevitably, given that so many promised interventions have been successfully achieved through collaborative action. But these relationships must be continuously strengthened and the conversations sustained if we are to build resilient communities into the future. Opportunities for further collaboration with the community will ensure that interventions are sustained over the longer term, for example, by affecting a community-led and officially sanctioned monitoring system. The building of trust and partnerships takes a great deal of time and effort, but is critical to effective engagement and enduring outcomes.

## Conclusion

The co-production of information through collaborative engagement is an inherently more democratic form of knowledge creation and knowledge transfer (Vogel et al. 2016). Drawing on the insights and understandings of ‘everyday specialists’ (Klein 2004), participatory processes may stimulate creative and alternative imaginaries (Vogel et al. 2016). This has been illustrated in the case of the Wallacedene TRA informal settlement in Cape Town, South Africa, where as a result of a collaborative risk assessment exercise that delivered tangible results, the TRA community began to imagine another reality, recognising their capacity to redefine and reshape the space in which they live through their own agency.

This article has demonstrated how a CRA process can effectively create entry points for collaborative dialogue, facilitating multi-stakeholder engagement around key development issues related to risk reduction. Despite their light touch, the CRA training sessions have been shown to achieve significant developmental gains, whilst limiting the kinds of disturbances that have been noted in larger projects that anticipate more measurable outcomes (Winkler 2013). But whilst the integration of endogenous and expert forms of knowledge is critical for deriving tangible developmental outcomes (Mercer et al. 2010), the CRA has also been found to stimulate less tangible benefits, engendering ongoing debate and continued community reflection around local issues, with the potential to catalyse further action.

## Footnotes

1 A similar exercise undertaken in another informal settlement failed during the implementation stage when role players from the City, who had supported the intervention and were instrumental in its planning and technical design, were not authorised to action the planned intervention.
